# Developmental temperature, more than long‐term evolution, defines thermal tolerance in an estuarine copepod

**DOI:** 10.1002/ece3.10995

**Published:** 2024-02-20

**Authors:** Lauren Ashlock, Chelsea Darwin, Jessica Crooker, James deMayo, Hans G. Dam, Melissa Pespeni

**Affiliations:** ^1^ Department of Biology University of Vermont Burlington Vermont USA; ^2^ Department of Marine Sciences University of Connecticut Groton Connecticut USA

**Keywords:** adaptation, climate change, copepod, plasticity, salinity tolerance, thermal tolerance

## Abstract

Climate change is resulting in increasing ocean temperatures and salinity variability, particularly in estuarine environments. Tolerance of temperature and salinity change interact and thus may impact organismal resilience. Populations can respond to multiple stressors in the short‐term (i.e., plasticity) or over longer timescales (i.e., adaptation). However, little is known about the short‐ or long‐term effects of elevated temperature on the tolerance of acute temperature and salinity changes. Here, we characterized the response of the near‐shore and estuarine copepod, *Acartia tonsa*, to temperature and salinity stress. Copepods originated from one of two sets of replicated >40 generation‐old temperature‐adapted lines: ambient (AM, 18°C) and ocean warming (OW, 22°C). Copepods from these lines were subjected to one and three generations at the reciprocal temperature. Copepods from all treatments were then assessed for differences in acute temperature and salinity tolerance. Development (one generation), three generations, and >40 generations of warming increased thermal tolerance compared to Ambient conditions, with development in OW resulting in equal thermal tolerance to three and >40 generations of OW. Strikingly, developmental OW and >40 generations of OW had no effect on low salinity tolerance relative to ambient. By contrast, when environmental salinity was reduced first, copepods had lower thermal tolerances. These results highlight the critical role for plasticity in the copepod climate response and suggest that salinity variability may reduce copepod tolerance to subsequent warming.

## Graphical abstract

Copepods collected in the Long Island Sound were exposed to two different temperature conditions, ambient (18°C) and ocean warming (22°C). Copepods were in experimental conditions for one, three, and >40 generations before assessing temperature and salinity tolerance. Results highlight the importance of plasticity in defining the temperature response of copepods.

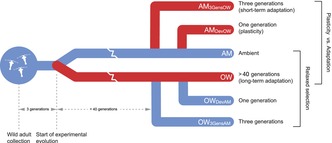

## 1 INTRODUCTION

Estuarine ecosystems are naturally dynamic, with anthropogenic impacts intensifying intrinsic variation in temperature and salinity (Hewitt et al., 2016). Mean water temperatures and marine heat waves are increasing with climate change (Bindoff et al., 2019; Harley et al., 2006; Scheffers et al., 2016). Additionally, climate‐driven changes in precipitation and storm events alter local salinity (Feher et al., 2023). Future salinity change will be location‐dependent, with wet regions predicted to decrease in salinity and dry regions predicted to increase in salinity (Skliris et al., 2014). With increasing fluctuations in temperature and salinity, it is important to understand how organisms tolerate these conditions and what tools they can use to respond to rapid anthropogenic change.

As the environment changes, populations can use plasticity and adaptation to respond. Plasticity is the ability of a genotype to exhibit multiple phenotypes in response to environmental change (Fordyce, 2006; Somero et al., 2017; West‐Eberhard, 2003; Whitman & Ananthakrishnan, 2009). Adaptation is genetically based phenotypic change driven by the process of selection that maximizes relative fitness in a given environment (Hartl, 2020; Hendry, 2016). Importantly, plasticity can result in new phenotypes within a generation, while adaptation acts across generations (Hartl, 2020; Somero et al., 2017). Understanding the relative roles of plasticity and adaptation in response to a changing climate will reveal the potential tolerance and vulnerabilities of marine organisms.

Estuaries are excellent ecosystems for understanding population‐level responses to a variety of stressors. Estuarine ecosystems are characterized by a temporally and spatially dynamic physical environment, with regular and stochastic variations in temperature and salinity (Moyle et al., 2010; Najjar et al., 2000). These complex ecosystems foster high productivity, and support large populations of fish, mammals, birds, and invertebrates (Harris et al., 2016; Moyle et al., 2010). Copepods are critical members of estuarine ecosystems, connecting primary producers to higher trophic levels, contributing to biogeochemical cycling, and providing a vital food source to forage fishes (Richardson, 2008; Steinberg & Landry, 2017). Copepods are also excellent sentinels of environmental change, as their short generation times allow them to closely mirror changing environmental conditions (Dam, 2013). Further, copepods are ideal models for laboratory experiments. They are easily cultured in a laboratory setting, and their short generation times facilitate their study across generations.

*Acartia tonsa* is a globally distributed, numerically dominant, estuarine copepod (Calliari et al., 2008; González, 1974; Johnson & Allen, 2012), which makes a suitable model for global change studies*. Acartia tonsa* is a generalist with respect to temperature and salinity, and is highly responsive and relatively robust to anthropogenic warming (Garzke et al., 2015; Rahlff et al., 2017; Rice et al., 2015; Rice & Stewart, 2016). Both plasticity and adaptation play a critical role in the *A. tonsa* response to temperature variation. Plasticity affects the thermal tolerance of *A. tonsa*, with populations that develop at higher temperatures having higher thermal tolerances (Sasaki & Dam, 2019). Additionally, *A. tonsa* subjected to 40 generations of experimental ocean warming (OW) show increased performance and fitness over time, indicating adaptation to OW (Dam et al., 2021). While the influence of plasticity and adaptation on *A. tonsa* thermal tolerance is characterized, the relative importance and potential limitations of plasticity and adaptation in responding to multiple climate change stressors remain unknown.

Climate change may leave *A. tonsa* vulnerable to additional stressors, particularly in estuaries where periods of extreme salinity fluctuation coincide with warm temperatures (Heilmayer et al., 2008; Tolley et al., 2005). In corals and the tidepool copepod, *Tigriopus californicus*, increased salinity can impart additional thermal tolerance (Denny & Dowd, 2022; Gegner et al., 2017), while low environmental salinities are associated with narrowing thermal performance curves and elevated expression of heat shock proteins in the copepod *Acartia tonsa* (Peck et al., 2015; Petkeviciute et al., 2015), and reduced thermal tolerance in *T. californicus* (Kelly et al., 2016). Investigating the influence of temperature on salinity tolerance, and salinity on temperature tolerance in a widespread and numerically dominant copepod like *A. tonsa* will reveal the sensitivity of this impactful organism to future climate change.

Here, we measured the impact of developmental (one generation), short‐term (three generations), and long‐term (>40 generations) multigenerational exposure to ocean warming (OW) on acute temperature and salinity tolerance. We asked four questions with this experimental design: (1) How does ocean warming impact thermal tolerance within and among generations? (2) Is thermal tolerance lost when ocean warming animals return to ambient conditions? (3) Does ocean warming impact salinity tolerance? (4) Does reduced salinity impact thermal tolerance? We predicted developmental exposure, short‐term adaptation, and long‐term adaptation to OW would increase copepod tolerance to acute heat stress. Specifically, we anticipated that tolerance to acute heat stress would increase proportionally to the number of generations exposed to OW. We predicted that thermal tolerance would be lost with a return of ocean warming line animals to ambient conditions, suggesting a cost to maintaining high thermal tolerance. Lastly, we predicted that any experimental exposure to OW would reduce copepod tolerance to acute salinity stress and, similarly, that low salinity exposure would reduce thermal tolerance.

## 2 MATERIALS AND METHODS

### 2.1 Collection and culture

**FIGURE 1:**
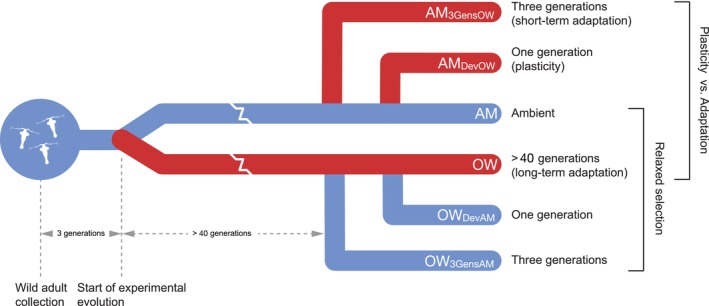
Schematic of experimental design. Blue lines represent AM temperature conditions (18°C) and red lines represent OW conditions (22°C). Plasticity versus adaptation bracket indicates the treatment groups necessary to compare the relative roles of plasticity and adaptation in the copepod climate response. The relaxed selection bracket indicates the treatment groups necessary to identify a potential loss in thermal tolerance. Abbreviations represent as follows: AM_3GensOW_, ambient animals that spent three generations at ocean warming; AM_DevOW_, ambient animals that developed at ocean warming; AM, ambient animals; OW, ocean warming animals; OW_DevAM_, ocean warming animals that developed at ambient; OW_3GensAM_, ocean warming animals that spent three generations at ambient.

Copepods in this study were derived from a previous experimental evolution project (Dam et al., 2021). Briefly, adult *A. tonsa* were collected from the Long Island Sound (41.3° N, 72.0° W; Groton, CT, USA) in June 2016 using a 400 μm plankton net with a solid cod end. Animals were raised as stock cultures at the University of Connecticut, Avery Point campus for at least three generations (~45 days) before splitting them into two treatments (ambient (AM): 18°C, ocean warming (OW): 22°C), with four replicates per treatment (Figure 1). The experimental conditions began in July 2017 and animals were transferred to UVM after ~40 generations in the experimental conditions in January 2019. Each stock culture was started with 160 female and 80 male adult copepods. Stock cultures yielded an average of 7173 eggs to seed each replicate culture. Replicates were fed ad libitum with a mixture of the phytoplankters *Rhodomonas* sp., *Tetraselmis* sp., and *Thalassiosira weissflogii*. All replicates were maintained at a salinity ranging from 31 to 36 ppt. Copepod and algal cultures were maintained on a 12:12 light dark cycle. Algal cultures were intentionally raised at AM temperature (18°C) to avoid potential changes in nutritional content if they were cultured at OW conditions.

After >40 generations, animals from all eight replicates (two treatments, with four replicates per treatment) were transferred to the University of Vermont (UVM) in January 2019. Organisms were transported at 18°C for 5 h and returned to their respective temperature conditions upon arrival at UVM. Light and food conditions were maintained the same as at Avery Point.

Additional transplant lines were created at UVM to test our hypotheses. Transplant cultures were created by setting aside all adults from each replicate to lay eggs for 48 h. All eggs produced by each replicate were separated in half by volume. Half of the eggs remained in the home condition, while the other half were used to seed a new culture at the reciprocal condition. Transplant cultures included AM line eggs that spent three generations in OW conditions (AM_3GensOW_) and OW line eggs that spent three generations in AM conditions (OW_3GensAM_). This process was repeated to create an additional set of transplant cultures:AM line eggs that developed in OW conditions (AM_DevOW_) and OW line eggs that developed in AM conditions (OW_DevAM_) (see Appendix I: Figure S1 for a detailed schematic).

### 2.2 Assess the effect of ocean warming on thermal tolerance within and among generations

To test the effect of ocean warming on thermal tolerance, we compared upper lethal temperatures (ULT) among four groups: ambient line animals, ocean warming line animals, ambient line animals that developed at ocean warming, and ambient line animals that spent three generations at ocean warming. To assess ULT, adult individuals were placed in their own individual well of a 12‐well plate with water at their treatment temperature, food replete conditions, and 30 ppt salinity. Adults were isolated from the culture the day prior to the assay and allowed to adjust to the plate wells at their respective treatment temperatures overnight. The following day, plates were removed from incubators and plates from both treatments were equilibrated to room temperature (~22°C). Plates were then sealed with parafilm and placed in a water bath where the temperature ramped from 22 to 34°C over 60 min (0.2°C/min), unmonitored. These temperatures were unmonitored because previous ULT assays in our laboratory demonstrated that they are sublethal to *A. tonsa* from the Long Island Sound (Appendix I: Figure S2). Temperature was then ramped at a rate of 0.0140°C/min. Copepods were assessed for survival (no movement for 10 s after disturbing the water in the well) at every 0.5°C until no surviving copepods remained. ULT was assessed for 12 individuals from each of the AM and AM_3GensOW_ conditions (four individuals per replicate × three replicates), 16 individuals of the AM_DevOW_ condition (four individuals per replicate × four replicates), and 15 individuals of the OW condition (four individuals per replicate × three replicates, and three individuals per replicate x one replicate).

### 2.3 Test for loss of thermal tolerance when ocean warming animals return to ambient conditions

To assess if thermal tolerance is lost after a return to ambient conditions, we compared the ULT between four groups: ocean warming animals, ambient animals, ocean warming animals that developed at ambient conditions, and ocean warming animals that spent three generations at ambient conditions. ULT was measured in the ocean warming line and ambient line animals as described above in Section 2.2, ULT was assessed for 12 individuals each from the OW_3GensAM_ and OW_DevAM_ conditions (four individuals per replicate × three replicates; Appendix I: Figure S1).

### 2.4 Test the effect of ocean warming on salinity tolerance

To examine the impact of ocean warming on salinity tolerance, we compared the hyposalinity tolerance of three groups: ambient line animals, ambient line animals that developed at ocean warming, and ocean warming line animals. Lower lethal salinity (LLS) was assessed for a total of 44 adult copepods, 12 individuals in the AM condition (four individuals per replicate x three replicates), 16 individuals in the AM_DevOW_ condition (eight individuals per replicate x one replicate, and four individuals per replicate × two replicates), and 16 individuals in the OW condition (four individuals per replicate × four replicates; Appendix I: Figure S1). To conduct the LLS assay, copepods were placed in their own individual well in a 12‐well plate. Wells did not contain food and had a starting salinity of 30 ppt. Temperature was maintained the same as the respective treatment temperature conditions. Adults were isolated from the culture the day prior to the assay and allowed to adjust to the plate wells overnight. Adults started at 30 ppt, and then salinity was reduced stepwise by full water replacement every 30 min with target salinities of 30 to 20 ppt, 15, 10, 8, 6, 4, 2, and 0 ppt. Salinity was measured at each step with a refractometer, and endpoint observed salinity was recorded. Survival was monitored at every step. Preliminary experiments revealed limited mortality above 10 ppt salinity (Appendix I: Figure S3), therefore we focused on finer resolution increments at lower salinity levels.

### 2.5 Test the effect of low salinity exposure on thermal tolerance

To explore the relationship between temperature and salinity tolerance further, we reversed the order of events and measured the effect of low salinity acclimation on ULT. For these assessments, we quantified the ULT for 20 adult copepods (seven individuals x three salinity levels, with 15 ppt only having six individuals). For this assay, animals were acclimated to three sublethal salinity conditions: 30, 20, and 15 ppt. One‐third of adults started and remained at 30 ppt, another set was moved stepwise from 30 to 20 ppt after 30 min, and another set was moved from 20 to 15 ppt after an additional 30 min. Animals were maintained at 18°C throughout the assay. After 12 h of acclimation to the three salinity levels (with 100% survival), animals from all treatments were assessed for their ULT. ULT assays were performed at the respective acclimatized salinity.

### 2.6 Data analysis

All analyses were carried out in R version 4.2.2 (2022‐10‐31; R Core Team, 2022).

We assessed the normality of the data using the Shapiro–Wilk test from the R stats package (R Core Team, 2022) and tested for homogeneity of variance using the Levene test from the car package (Fox & Weisberg, 2019). Our results indicated that our data were not normally distributed and did not have equal variance across treatments. Therefore, differences between ULT and LLS were assessed using non‐parametric methods. We performed an additional Levene test to reveal potential differences in variance among treatments. This was done by calculating residuals, running an ANOVA using the R stats package, and then doing a post hoc test using Tukey's HSD in the R stats package (R Core Team, 2022). We used the nonparametric Aligned Rank Transform (ART) ANOVA in cases with more than one independent variable (Wobbrock et al., 2011). The ART ANOVA, and multifactor contrast tests, were performed using the R package ARTool (Elkin et al., 2021; Wobbrock et al., 2011). We used the Kruskal–Wallis test using the rcompanion package (Mangiafico, 2023) when there was only one independent variable. The Dunn test was used to test multiple pairwise comparisons after the Kruskal–Wallis test using the FSA package (Ogle et al., 2023) when applicable. To determine the effect of replicate on our dependent variables, we used the Kruskal–Wallis test. Replicate had no effect on ULT (*p* = .24) and was therefore not included in the model. In contrast, replicate had an effect on LLS (*p* < .001); thus, was included in the model as a random effect. In our experiment described in section 2.5, replicate was not recorded and therefore not incorporated in the model.

## 3 RESULTS

### 3.1 How does ocean warming impact thermal tolerance within and among generations?

**FIGURE 2:**
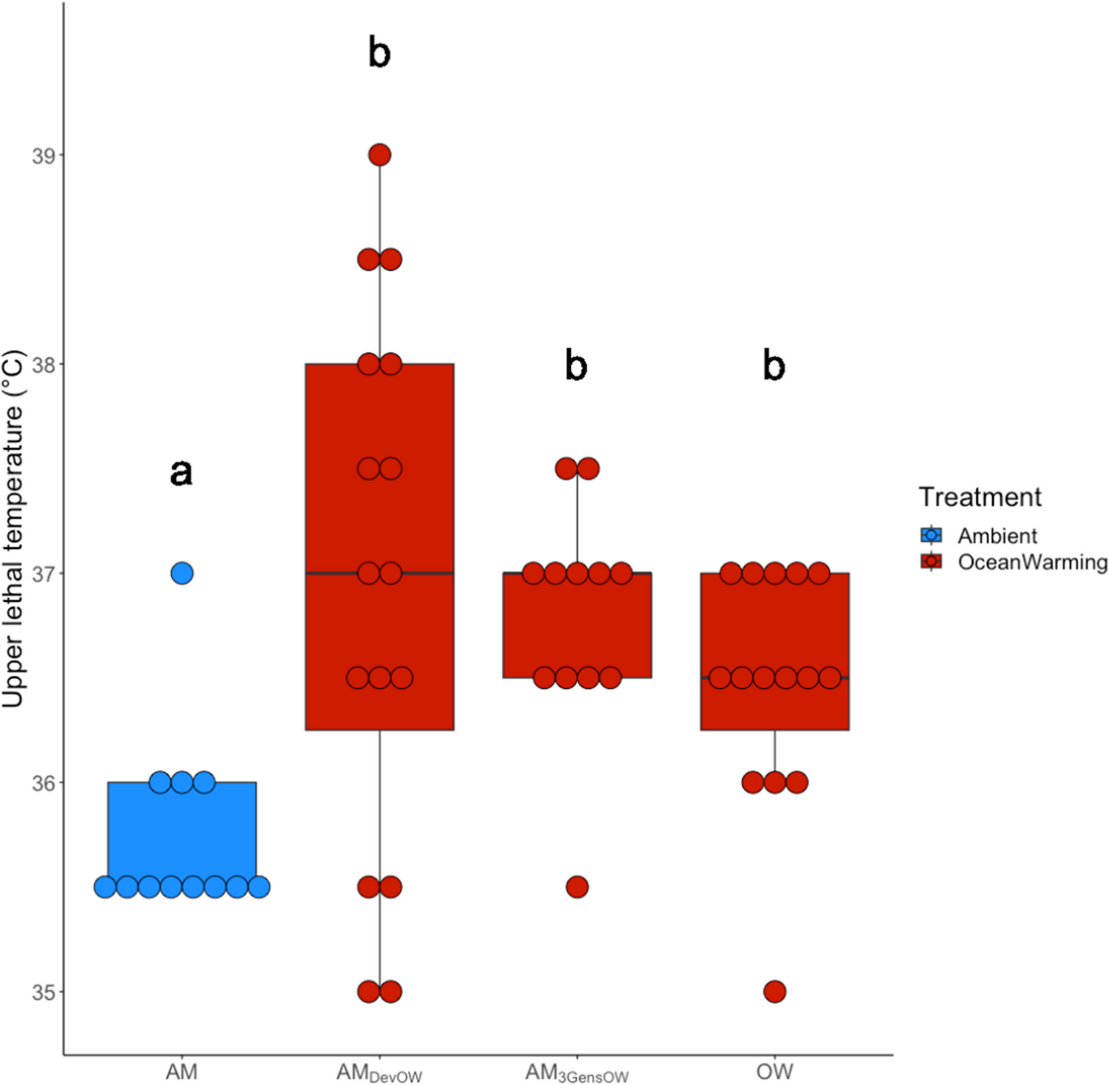
Upper lethal temperature (ULT) for ambient line animals moved into ocean warming conditions for one (AM_DevOW_), three (AM_3GensOW_), and >40 generations (OW). Dots represent individual copepods, each in their own well. Color denotes treatment temperature. Letters denote significance. AM, ambient animals; AM_DevOW_, ambient animals that developed at ocean warming; AM_3GensOW_, ambient animals that spent three generations at ocean warming; OW, ocean warming.

Increasing temperature to 22°C for one, three, or >40 generations resulted in increased thermal tolerance for copepods (Dunn test, *Z* = −3.65, −3.78, 2.85, *p*_*adj*_ = .002, .002, and .02, respectively; Figure 2). Importantly, there was no additional thermal tolerance gained by spending three generations or >40 generations at 22°C, compared to animals that just spent development at 22°C (Dunn test, *Z* = −0.39, *p*_adj_ = .69 (AM_3GensOW_), *Z* = −0.81, *p*_adj_ = .48 (OW); Figure 2). The similarity in thermal tolerance between animals that developed at 22°C and animals that spent >40 generations at 22° demonstrates that plasticity imparts the same thermal tolerance as >40 generations at 22°C. In addition, ambient line animals that developed at 22°C had greater variance in ULT compared to all other treatment groups (Levene Test, *p*_adj_ = .001 (AM), 0.009 (AM_3GensOW_), and 0.004 (OW)), suggesting that elevated temperature during development reveals a diversity of ULT phenotypes that are lost over subsequent generations.

### 3.2 Is thermal tolerance lost when ocean warming animals return to ambient conditions?

**FIGURE 3:**
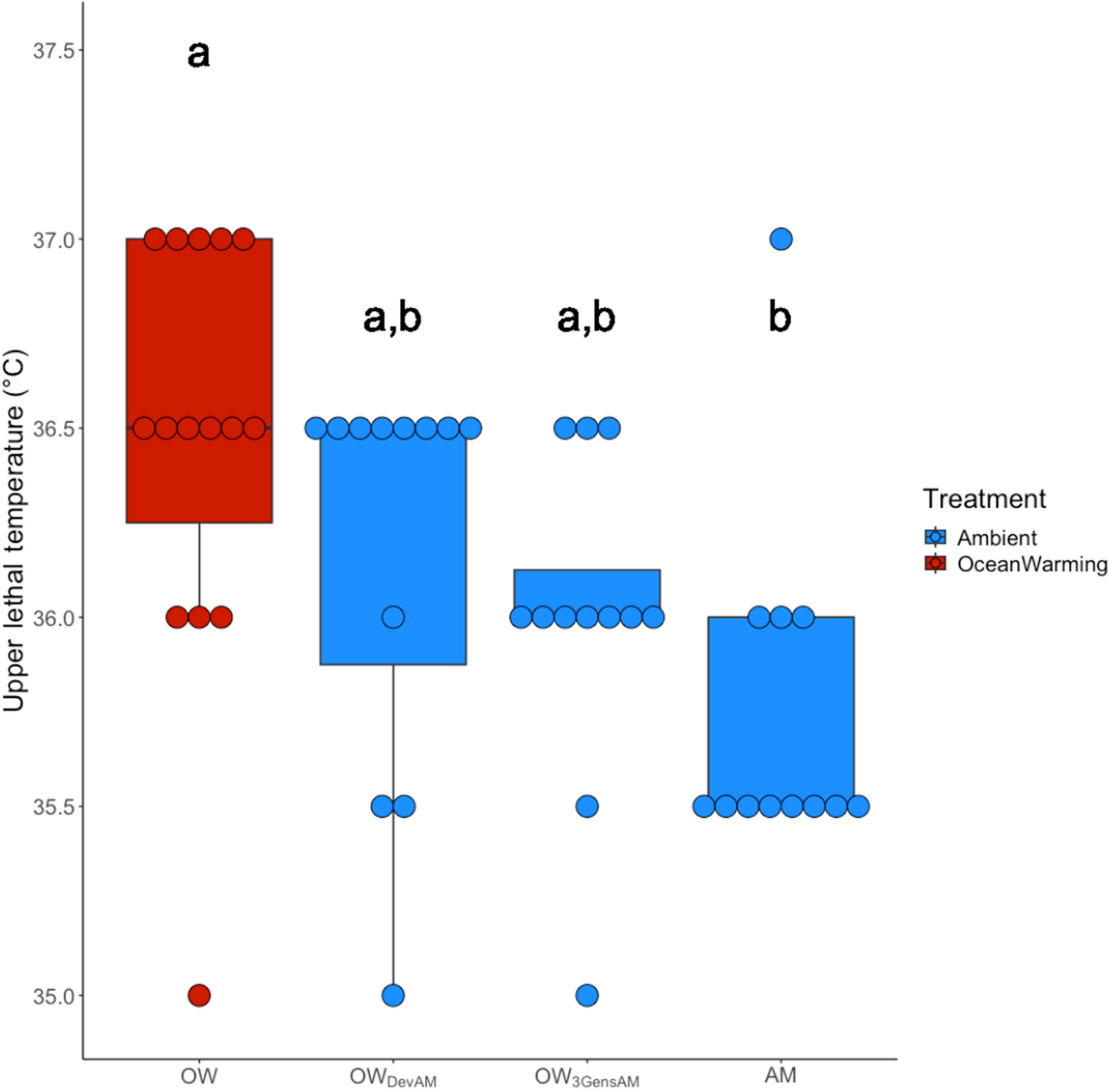
Upper lethal temperature (ULT) for ocean warming line animals moved into ambient conditions. Abbreviations from left to right: OW, ocean warming; OW_DevAM_, ocean warming line animals that developed at the ambient temperature; OW_3GensAM_, ocean warming line animals that spent three generations at the ambient temperature; and AM, ambient. Dots represent individual copepods, each in their own well. Color denotes treatment temperature. Letters denote significance. Note that ocean warming and ambient groups are the same data represented in Figure 2, presented separately for clarity of hypothesis testing.

To reveal any sustained benefits or costs of long‐term adaptation to ocean warming, animals from the OW line were transplanted into ambient conditions for one (OW_DevAM_) and three generations (OW_3GensAM_). For ocean warming line animals that developed at ambient temperatures and ocean warming line animals that spent three generations at ambient temperatures, there was no significant loss of thermal tolerance (Figure 3, Dunn test, *Z* = 1.17, 1.95, *p*_adj_ = .36 and .10, respectively). However, animals from both of these transplanted groups had intermediate thermal tolerances between the AM and OW line animals (Figure 3).

### 3.3 Does ocean warming impact salinity tolerance?

**FIGURE 4:**
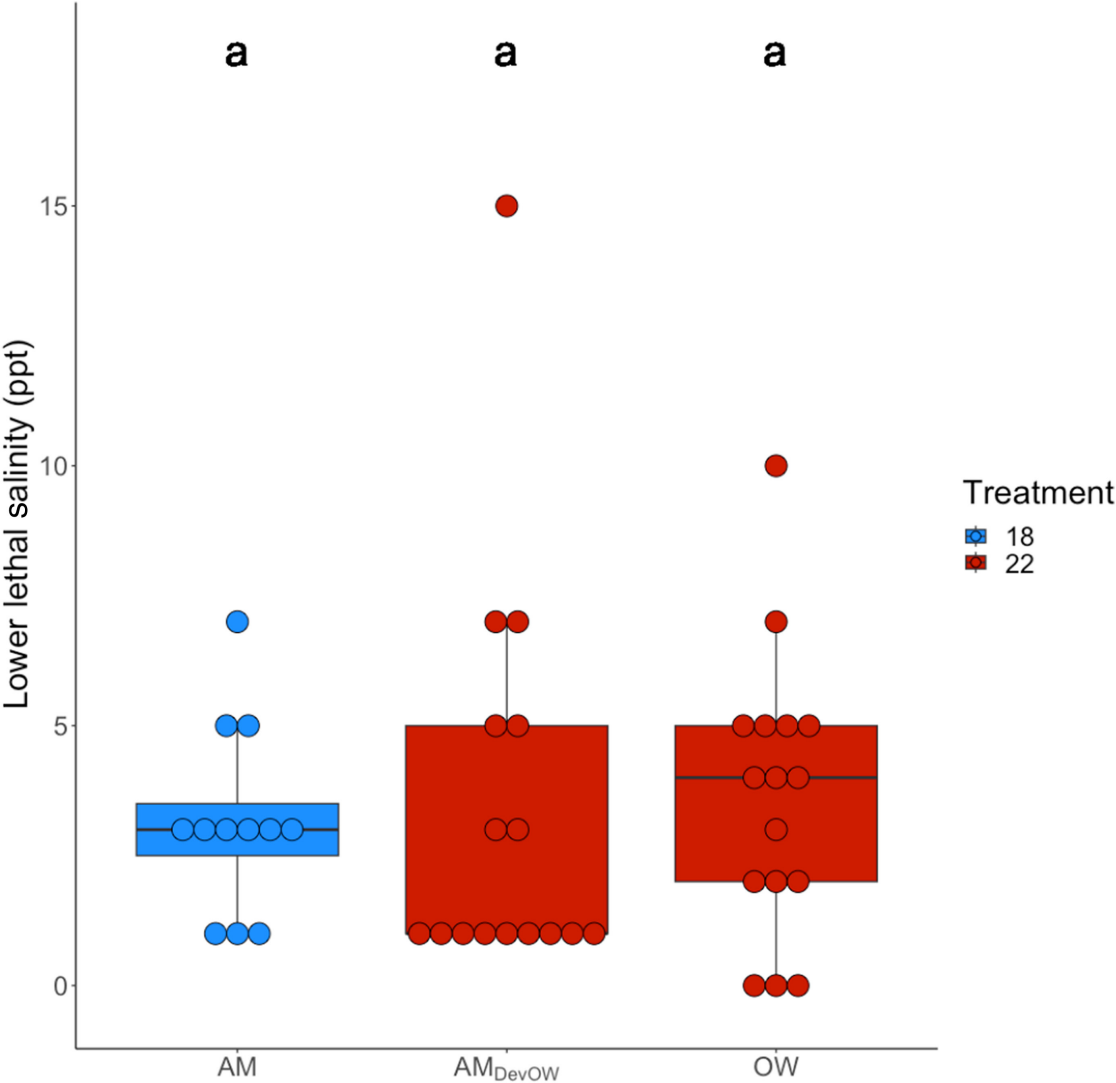
Lower lethal salinity (LLS) after one (AM_DevOW_) and >40‐generations at warming (OW). Dots represent individual copepods, each in their own well. Color denotes treatment temperature. Letters note significance.

To test for an effect of short‐ and long‐term ocean warming on LLS, we exposed ambient line animals, ambient line animals that developed at ocean warming, and ocean warming line animals to sequentially lower salinities. We found no effect of one or > 40 generations of ocean warming on low salinity tolerance relative to ambient line animals (ART ANOVA, *F* = 0.67, *p* = .52, Figure 4), suggesting no costs of elevated temperature on low salinity tolerance.

### 3.4 Does hyposalinity exposure impact thermal tolerance?

**FIGURE 5:**
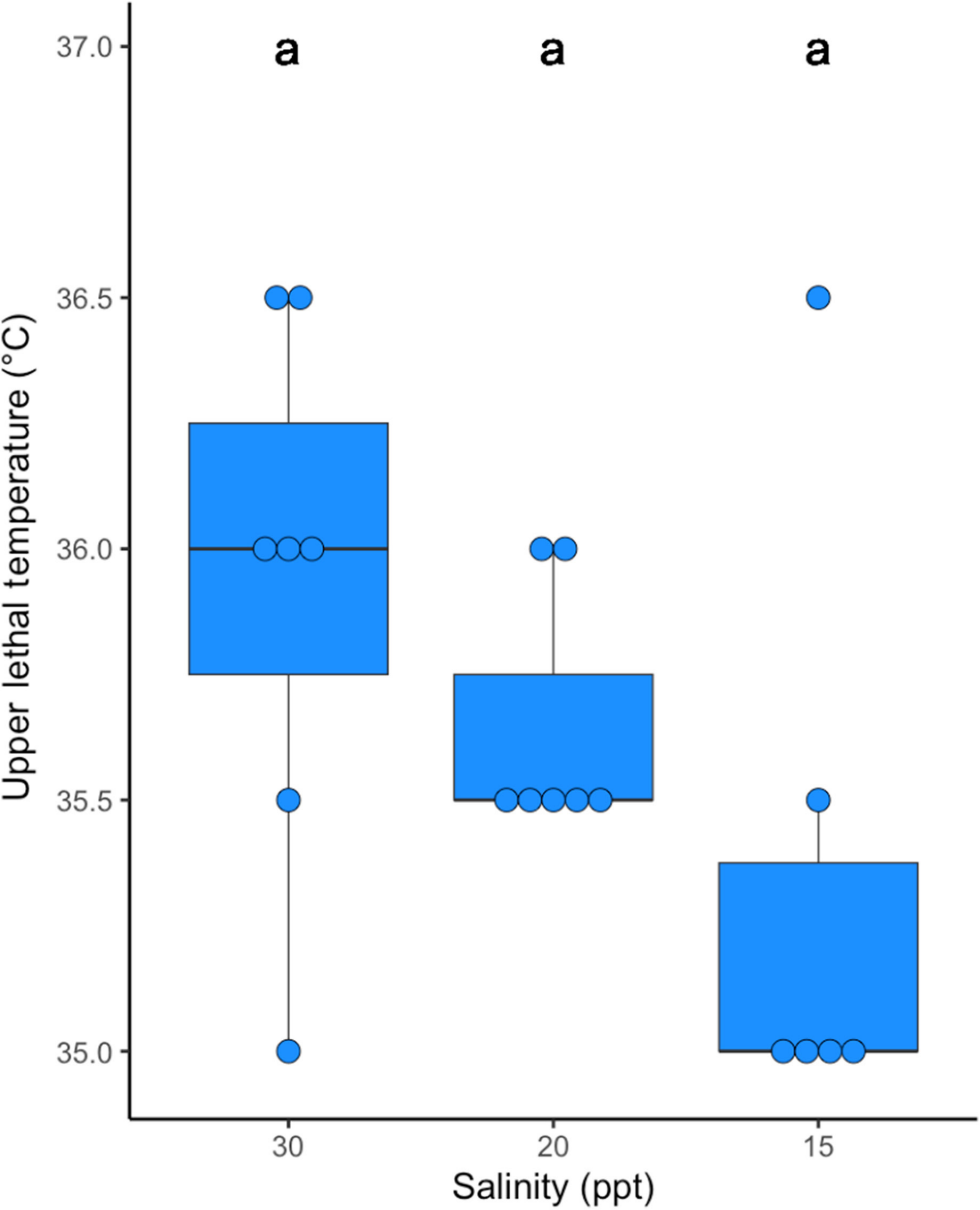
Upper lethal temperature (ULT) after sequential lowering of salinity for ambient line animals. Dots represent individual copepods, each in their own well. Letters above boxes denote significance.

To test if reducing environmental salinity affects thermal tolerance, we exposed individual copepods to decreasing salinity conditions and subsequently assayed them for their ULT. To do this, we sequentially lowered the salinity from 30, to 20, to 15 ppt for ambient line animals. Animals at all three salinities were tested for their ULT. Comparing among the salinity treatments, ULT was marginally higher for copepods that stayed at 30 ppt than copepods that were moved to 15 ppt (ART ANOVA contrasts *p* = .07). We found that 1 day at a lower salinity of 15 ppt reduced mean ULT of ambient line animals by 0.6°C (Figure 5).

## 4 DISCUSSION

Here, we examined the effect of short and long‐term ocean warming on acute temperature and salinity tolerance in the estuarine copepod *A. tonsa*. As predicted, warming for one, three, or >40 generations increased copepod tolerance to acute heat stress. However, thermal tolerance did not increase proportionally to the number of generations in elevated temperature conditions. Rather, copepods from ambient conditions that developed at ocean warming or spent three or >40 generations at ocean warming had the same mean thermal tolerance, indicating that plasticity imparts the same thermal tolerance as >40 generations of evolution in ocean warming conditions. Development in ocean warming also revealed phenotypic diversity in thermal tolerance in ambient line animals that was not visible in ambient conditions. This phenotypic diversity was presumably lost after three and >40 generations in ocean warming. Our prediction that warming would reduce copepod tolerance to acute salinity stress was not supported, with no duration of experimental warming affecting LLS. Alternatively, we did find evidence that decreasing environmental salinity can impact thermal tolerance, with exposure to a sequential decrease in salinity leading to animals with marginally lower thermal tolerances. These results suggest that increasingly dynamic salinity conditions in estuarine ecosystems may increase warming‐induced mortality in this critical copepod species. Our results also indicate that tolerance to one stressor is impacted by the occurrence of additional stressors. As oceans are multifaceted ecosystems with many concurrent changing variables, it is essential to consider how these environmental stressors interact to determine organismal tolerance.

### 4.1 Warming increases thermal tolerance within and among generations

We found that ambient line animals that developed at ocean warming had a higher thermal tolerance than ambient line animals that stayed at ambient, indicating the importance of plasticity in *A. tonsa* thermal tolerance. An additional three and >40 generations at 22°C did not further improve thermal tolerance beyond development in ocean warming conditions. This differs from *A. tonsa* LD_50_ after experimental evolution to warming, which continuously improved across 40 generations (Sasaki & Dam, 2021a). Differences here may be due to differences in the thermal tolerance metric assessed. Sasaki & Dam, 2021a assessed LD_50_, the temperature at which 50% of the population dies, whereas in this study we assessed Upper Lethal Temperature (ULT), the temperature at which each individual copepod dies. ULT represents a hard physiological limit and is likely more constrained than LD_50._ The importance of plasticity in *A. tonsa*, demonstrated by our results, aligns well with existing research that demonstrates relatively high plasticity in the *A. tonsa* response to elevated temperature conditions (Garzke et al., 2015; Rahlff et al., 2017; Rice & Stewart, 2016; Sasaki & Dam, 2019). Our results also corroborate research in *Daphnia* sp. demonstrating that acclimation temperature has a larger impact on thermal tolerance than local adaptation (Yampolsky et al., 2014). Additionally, studies have found that plasticity can play a larger role than genetic variation in determining temperature tolerance in *Drosophila melanogaster* (Ayrinhac et al., 2004; Hoffmann et al., 2005). Together, these results indicate that plasticity plays a critical role in thermal tolerance.

Plasticity may be dampened, however, after adaptation to warming or multiple stressors. Heat tolerance selection in the tidepool copepod, *Tigriopus californicus*, resulted in reduced phenotypic and gene expression plasticity (Kelly et al., 2017). Additionally, after long‐term adaptation to concurrent warming and increased pCO2, *A. tonsa* exhibited reduced transcriptional plasticity (Brennan, DeMayo, Dam, Finiguerra, Baumann, & Pespeni, 2022). Adaptation to the same combination of stressors reduced thermal tolerance plasticity in *A. tonsa* (deMayo et al., 2021). More broadly, research demonstrates that ectotherms across fresh water, salt water, and terrestrial habitats exhibit a trade‐off between thermal tolerance plasticity and upper thermal limits (Barley et al., 2021, Sasaki & Dam, 2021b). This has in some cases been referred to as a concrete ceiling to thermal tolerance (Sandblom et al., 2016), which can leave organisms vulnerable to continuous warming and unpredictable temperature variability.

We found that variability in ULT differed across treatments, indicating that introduction to novel environments or changing temperature may increase trait variability. Interestingly, one generation of development in warming revealed a wider range of ULT values than other treatment groups (Figure 2). This result supports theory and other empirical studies that suggest novel environments may disrupt organism homeostasis (Badyaev, 2005) and reveal trait variability that is otherwise hidden (Badyaev, 2005; Salinas et al., 2019). Importantly, treatment temperatures were static. Therefore, copepods in the three and > 40‐generation warming treatments experienced no temperature variability during the experimental generations prior to thermal tolerance assessment. This is distinct from the developmental treatment that experienced a temperature change from 18°C to 22°C during early development. Differences in trait variability may be due to this difference in exposure to temperature variability. Future work in this system should vary the amplitude and predictability of treatment temperature (Bitter et al., 2021), to elucidate the influence of these factors on plasticity in thermal tolerance. Additionally, such experiments could test if variability that is lost beyond one generation at warming is due to constant temperature conditions in the laboratory, or if there are other costs to maintaining high thermal tolerances.

### 4.2 Is thermal tolerance lost when returning ocean warming line animals to ambient conditions?

Our results indicate a cost of adaptation to ocean warming. For example, the ambient line that developed at ocean warming conditions exhibited the highest observed thermal tolerances in our study (ULT > 38°C) that were no longer observed after three and > 40 generations at 22°C. Additionally, we see decreasing thermal tolerances in ocean warming line animals that developed at ambient and ocean warming line animals that spent three generations at ambient relative to animals that spent >40 generations at ocean warming conditions, suggesting that maintaining high thermal tolerances is potentially costly under ambient conditions. There are established inherent costs of animal exposure to elevated or stressful temperatures, such as the increased need for heat shock proteins, ubiquitination of denatured proteins, and restructuring of cell membranes to maintain ion homeostasis (Somero, 2002). Without exposure to chronic elevated temperature, maintaining these responses may come at too high a cost. Additionally, as mentioned earlier, maintaining a high thermal tolerance may come at a cost of being able to maintain thermal tolerance plasticity (Barley et al., 2021, Sasaki & Dam, 2021b). Therefore, it may be more beneficial for animals held at ambient conditions and animals that had experienced multiple temperatures during their development to maintain thermal tolerance plasticity rather than maintain higher thermal tolerance.

Our results suggest the developmental environment influences ULT. We observed an intermediate thermal tolerance phenotype in ocean warming animals that developed at ambient and ocean warming animals that spent three generations at ambient, between ocean warming and ambient. This loss of thermal tolerance after one and three generations in ambient conditions may indicate relaxed selection, where the removal of a selective force leads to trait loss (Lahti et al., 2009). Importantly, the developmental environment plays a critical role in defining thermal tolerance (Ayrinhac et al., 2004; Hoffmann et al., 2005; Sasaki & Dam, 2019; Schaefer & Ryan, 2006). Therefore, lower thermal tolerances may be due to acclimation to a lower developmental temperature. Despite this resultant loss of thermal tolerance, it is important to remember that thermal tolerance was gained within one generation at ocean warming for the ambient line animals. Therefore, even if extended periods of relaxed temperature selection occur, *A. tonsa* is likely capable of quickly regaining thermal tolerance during periods of warming.

### 4.3 Order of events matters for salinity and thermal tolerance

Counter to our initial hypothesis, there was no effect of ocean warming on low salinity tolerance. Our results align well with empirical studies done in the intertidal copepod *Tigriopus californicus* that revealed selection for increased heat tolerance did not impact salinity tolerance (Kelly et al., 2016). In contrast, when the order of events was reversed, we found that exposure to low salinity conditions resulted in lower thermal tolerances for *A. tonsa*, similar to findings in *T. californicus* (Kelly et al., 2016). The authors hypothesize this may be due to competing energetic demands between osmoregulation and responding to increasing temperatures. These results together are particularly important, because *T. californicus* and *A. tonsa* are hardy species that experience regular salinity and temperature fluctuations, yet for both short‐term hyposalinity conditions reduce thermal tolerance. Additionally, in oysters (*Crassostrea virginica*) found in the Gulf of Mexico, low salinity events that coincide with the warm season cause increased mortality and reduced growth and recruitment, compared to salinity events that happen in cooler months (La Peyre et al., 2013). Taken together, these results indicate that even euryhaline species are vulnerable to simultaneous salinity and temperature fluctuations. These conditions are environmentally relevant to low‐latitude populations of *A. tonsa*, as summer corresponds with the wet season in the Gulf of Mexico coastal estuaries. Therefore, copepods and other estuarine and nearshore animals are exposed to periods of extreme salinity fluctuations and warm temperatures at the same time (Heilmayer et al., 2008; Tolley et al., 2005). With continued climate change, we expect more precipitation extremes and marine heat waves (Frölicher et al., 2018; Oliver et al., 2018; Singh et al., 2013). Such conditions could have negative implications for *A. tonsa* and other copepods that are less tolerant of temperature and salinity fluctuations.

### 4.4 Potential experimental limitations

There are some limitations within our experimental design that could impact results.

One limitation is that not all original experimentally evolved replicate lines were sampled for all assays (See Appendix I: Figure S1 for experimental details). For ULT, we found that replicate had no effect (*p* = .24); therefore, sampling three out of four replicate lines is unlikely to impact our results. However, replicate did have a significant effect on LLS (*p* < .001). While we included replicate in the model for LLS, it is possible that we could have observed a significant difference among temperature treatment groups if all replicate lines were included. However, given the consistent, low salinity tolerance, this is unlikely. In contrast, that food was not used in the LLS assay, because we focused on relative differences in salinity tolerance between treatments, could have affected results. Copepods with higher resource availability perform better at suboptimal salinities (Hammock et al., 2016; Rippingale & Hodgkin, 1977), although this is not a universal feature in copepods (Van Someren Gréve et al., 2020). Therefore, salinity tolerances could have been higher under food replete conditions obscuring the differences we were able to observe. Lastly, changes in ULT after three generations in ocean warming could be due to plasticity or adaptation. Results from related studies in *A. tonsa* demonstrate that fitness declined after one generation in warming conditions but improved by three generations (Dam et al., 2021). In addition, there were consistent, directional changes in allele frequencies among replicates over the span of 40 generations and changes were in genes related to cellular homeostasis, development, and stress response (Brennan, deMayo, Dam, Finiguerra, Baumann, Buffalo, et al., 2022; Brennan, deMayo, Dam, Finiguerra, Baumann, & Pespeni, 2022). Both studies show rapid adaptive capacity in *A. tonsa* to warming. Combined with the present results, these studies highlight the importance of developmental plasticity and suggest that both plasticity and adaptation play a role in shaping the thermal tolerance phenotype when copepods experience ocean warming.

## 5 CONCLUSION

We demonstrated the important contribution of plasticity in determining copepod thermal tolerance. Additionally, we found that environmental salinity reductions of 15 ppt resulted in a decrease in ULT by 0.6°C in ambient line animals. These salinity reductions are within the range already experienced by *A. tonsa* in the Long Island Sound. Therefore, our results indicate that *A. tonsa* in this region may experience increased mortality with increasing variation in temperature and salinity, suggesting that other less tolerant species may experience more severe consequences of these two shifting environmental variables. This motivates further exploration of the influence of temperature and salinity on the survival and fitness of additional marine ectotherms.

## AUTHOR CONTRIBUTIONS

**Lauren Ashlock:** Conceptualization (equal); formal analysis (equal); methodology (equal); visualization (equal); writing – original draft (equal). **Jessica Crooker:** Investigation (equal); visualization (equal); writing – review and editing (equal). **Chelsea Darwin:** Investigation (equal); visualization (equal); writing – review and editing (equal). **James deMayo:** Resources (equal); writing – review and editing (equal). **Hans G. Dam:** Funding acquisition (equal); methodology (equal); resources (equal); supervision (equal); writing – review and editing (equal). **Melissa Pespeni:** Conceptualization (equal); funding acquisition (equal); project administration (equal); resources (equal); supervision (equal); writing – review and editing (equal).

## CONFLICT OF INTEREST STATEMENT

The authors declare no conflicts of interest.

## Supporting information


Figure S1.

Figure S2.

Figure S3.

## Data Availability

Upper lethal temperature and lower lethal salinity data can be found on Dryad (https://datadryad.org/stash/share/LAsbEZB649wm0cEe4Q3IbMJ_QTnLBhAHjqlZ2PQwIpE) and R scripts used to analyze the data can be found at https://github.com/lashlock/Developmental‐Temperature‐more‐than‐Long‐Term‐Evolution‐Defines‐Thermal‐Tolerance.
