# Supplementary figures and images for: Developmental temperature, more than long‐term evolution, defines thermal tolerance in an estuarine copepod
Source: Ecol Evol. 2024 Feb 20;14(2):e10995. doi: 10.1002/ece3.10995 (PMC10877657; doi:10.1002/ece3.10995)

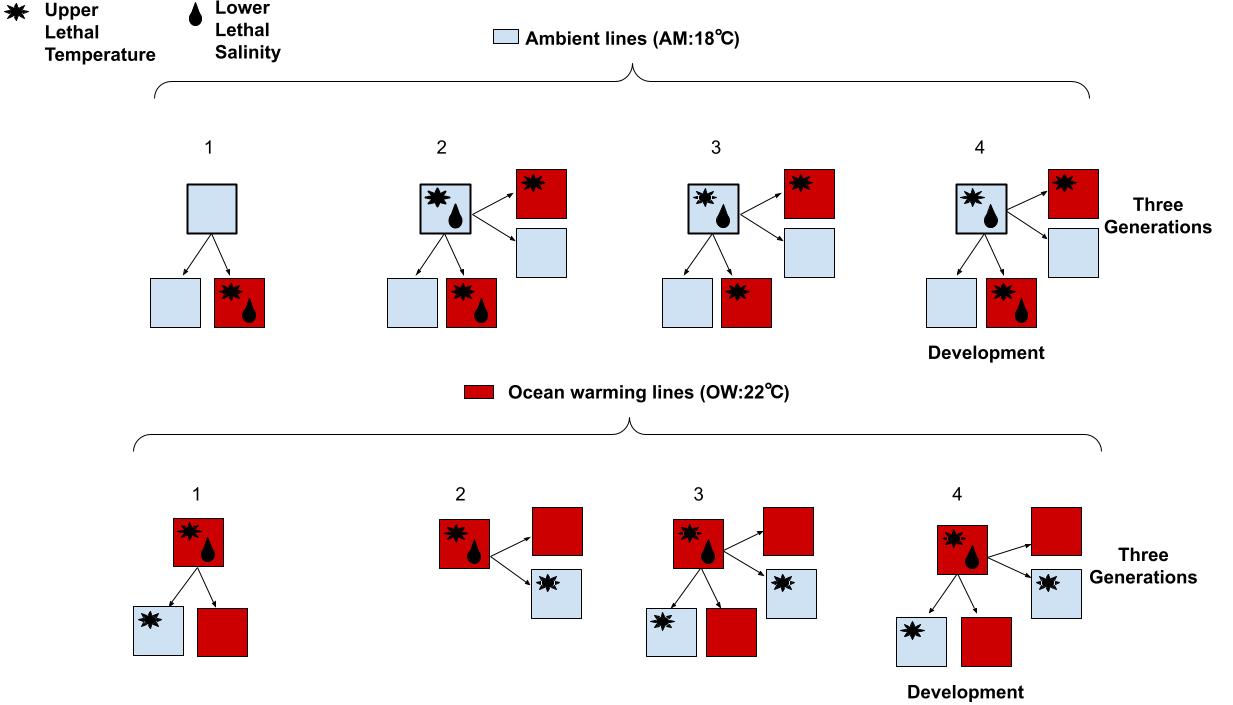

Supplement: Supplementary file 1 — Figure S1. [file ECE3-14-e10995-s002.jpg]

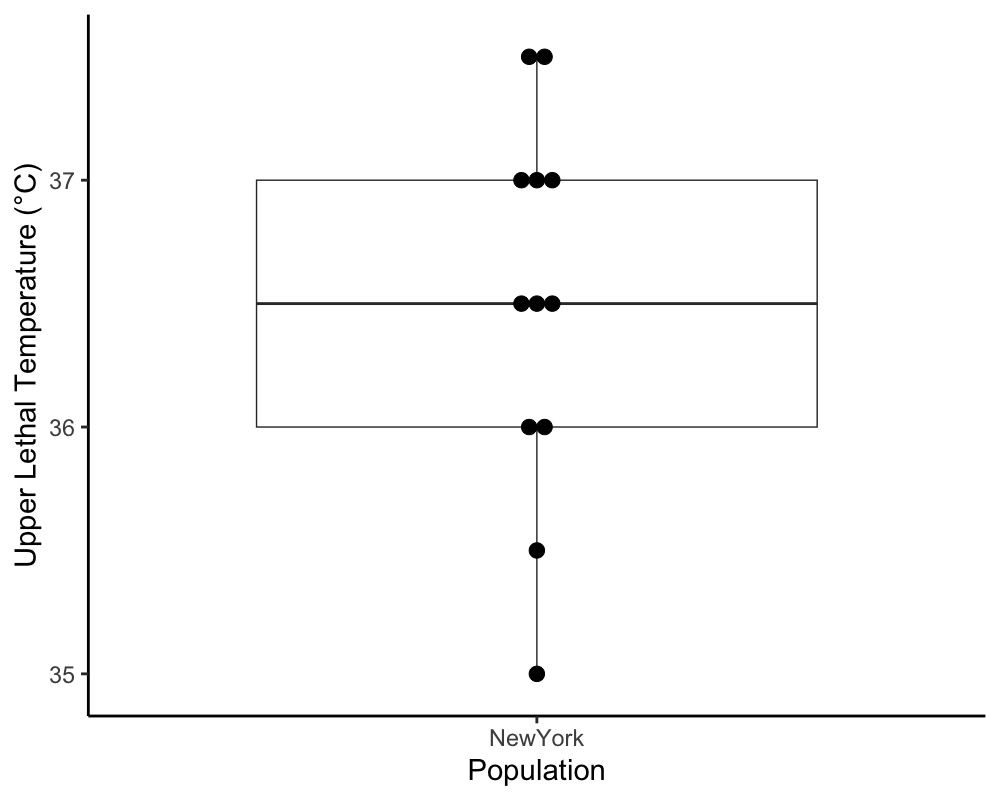

Supplement: Supplementary file 2 — Figure S2. [file ECE3-14-e10995-s001.jpeg]

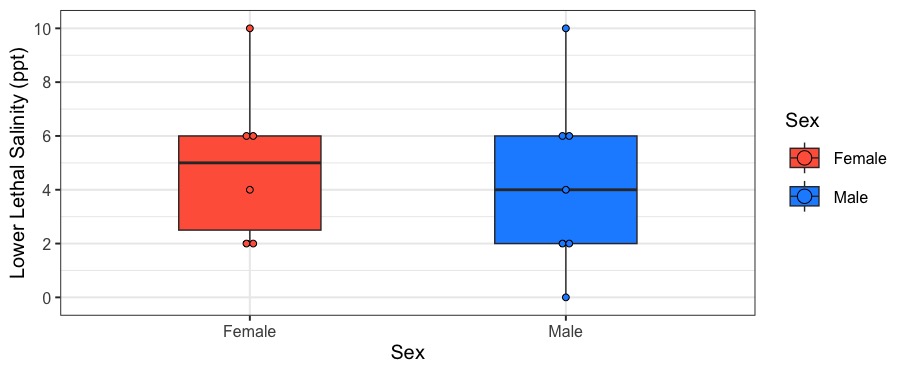

Supplement: Supplementary file 3 — Figure S3. [file ECE3-14-e10995-s003.jpeg]
